# The Pathway of Female Couples in a Fertility Clinic

**DOI:** 10.1055/s-0042-1744444

**Published:** 2022-06-03

**Authors:** Pedro Brandão, Nathan Ceschin, Victor Hugo Gómez

**Affiliations:** 1 Department of Reproductive Medicine, Instituto Valenciano de Infertilidad, IVIRMA Global Valencia, Valencia, Spain; 2 Faculty of Medicine, University of Porto, Porto, Portugal; 3 Department of Reproductive Medicine, Feliccità Instituto de Fertilidade, Curitiba, PR, Brazil

**Keywords:** donor conception, fertilization in vitro, homosexuality, reproductive techniques, assisted, ROPA, concepção do doador, fertilização in vitro, homossexualidade, técnicas reprodutivas, assistido, ROPA

## Abstract

**Objective** The present study aims to describe the main characteristics of female couples resorting to a fertility clinic, to understand whether these patients have clear previous plans concerning procreation and how they end up completing their family planning, and to briefly describe the main outcomes of the *recepción de ovocitos de pareja* (ROPA, in the Spanish acronym: in English, reception of partner's oocytes) method.

**Methods** This is a descriptive retrospective study of the pathway and outcomes of female couples in a fertility clinic during a 2-year period.

**Results** A total of 129 couples were treated. Only one third of the couples had no condition potentially affecting fertility or advanced age. Most couples were decided to undergo artificial insemination or *in vitro* fertilization and the majority kept their plans, as opposed to 38% of the couples who decided to the ROPA method (lesbian shared *in vitro* fertilization) who changed plans. Live birth rates per treatment (including frozen embryo transfers) for artificial insemination, 58% for in vitro fertilization, 80% for treatments with donated oocytes or embryos, and 79% for ROPA. Four in five couples achieved live births.

**Conclusion** The present study highlights the importance of a thorough medical workup in same-sex couples resorting to assisted reproduction. Despite the higher-than-expected rates of fertility disorders, the outcomes were good. Most couples end up in a single parented method. Furthermore, the results of the ROPA method are reassuring.

## Resumo

**Objetivo** O presente estudo tem como objetivo descrever as principais características dos casais femininos que recorrem a uma clínica de fertilidade, perceber se estas pacientes têm planos prévios claros sobre a procriação, como acabam por completar o seu planejamento familiar e descrever sucintamente os principais resultados do método fertilização *in vitro* compartilhada lésbica (ROPA, na sigla em espanhol).

**Métodos** Trata-se de um estudo retrospectivo descritivo da trajetória e dos resultados de casais femininos em uma clínica de fertilidade durante um período de 2 anos.

**Resultados** Um total de 129 casais foram tratados. Apenas um terço dos casais não apresentava nenhuma condição que afetasse potencialmente a fertilidade ou idade avançada. A maioria dos casais optou pela inseminação artificial ou fertilização *in vitro* e a maioria manteve seus planos, ao contrário dos 38% dos casais que decidiram se submeter ao método ROPA que mudaram de planos. As taxas de nascidos vivos por tratamento (incluindo transferências de embriões congelados) – 22% para inseminação artificial, 58% para fertilização *in vitro* , 80% para tratamentos com oócitos ou embriões doados e 79% para ROPA. Quatro em cada cinco casais conseguiram nascidos vivos.

**Conclusão** O presente estudo destaca a importância de um acompanhamento médico em casais femininos que recorrem à reprodução assistida. Apesar das taxas mais altas do que o esperado de distúrbios de fertilidade, os resultados foram bons. A maioria dos casais acaba em um método monoparental. Além disso, os resultados do método ROPA são tranquilizadores.

## Introduction

The impossibility of natural conception is one of the most challenging aspects within a gay couple. Nevertheless, medically assisted reproduction treatments (ARTs) have opened a variety of options for both female and male gay couples to procreate, including artificial insemination (AI) and in vitro fertilization (IVF) with donated sperm for female couples and surrogacy (partial or total, with donated oocytes) for male couples. 1 2 Furthermore, female couples have the possibility of sharing biological motherhood through a method called *recepción de ovocitos de pareja* (ROPA, in the Spanish acronym; in English, reception of partner's oocytes), or lesbian shared IVF. This consists of an IVF in which the oocytes of one of them (“donor” or “genetic mother”) are fertilized with donated sperm and the resulting embryo is transferred to the other member of the couple (“recipient” or “gestational mother”). 3 4 5 Artificial insemination and IVF with own or donated oocytes/embryos may also be called single parented methods (from a biological point of view), while the ROPA method is the only double-parented method.

Classically, assisted reproduction units were designed and prepared to receive and treat heterosexual couples. During the last decade, some units have been trying to adapt their activity to other kinds of patients, such as single patients and homosexual couples. Previous research has reported that homosexual couples tend to feel some lack of attention with their specific situation, including marketing policies directed to straight couples, decorations recalling heteronormative families, documents referring to a husband and wife, clinics specialized in fertility disorders, etc. 6 7 8

In contrary to heterosexual couples, female couples usually do not come to an assisted reproduction unit due to infertility. Thus, unless proven wrong, most of these couples are not expected to have any condition impairing fertility. Indeed, some of them come to IVF clinics with very well-defined plans concerning procreation, while others come to be informed for the first time about the treatments available and what would be most suitable for them. 9 10 11

However, it is important to notice that the majority of female couples has never tried natural conception, so their fertility potential is unknown. These patients may also suffer from any condition impairing fertility. Indeed, it is not uncommon to find an infertility factor during fertility workup or after unsuccessful fertility treatments. 12 This may be particularly difficult to deal with in patients with clear previous plans. Unlike heterosexual couples, it may not only imply changing the type of treatment, but also changing roles (who is going to be biological mother or who is going to be the gestational or genetic mother in case of ROPA). 13 14

Our work aims to describe general features of female couples who come to a fertility clinic, their intentions regarding assisted reproduction, the importance of medical workup and counseling, their pathway during assisted reproduction, and the outcomes of the reproductive treatments in this population, focusing on the ROPA method, the only treatment exclusive to female couples.

## Methods

This is a descriptive retrospective study. All female couples having their first appointment at IVIRMA Valencia, Spain, from January 2017 to December 2018 were included. The study protocol was approved by the local Institutional Ethical Review Board. During the first 2 appointments, basic medical workup is performed, including anamnesis, gynecologic examination, pelvic ultrasound, evaluation of ovarian reserve by dosing antimüllerian hormone (AMH) levels and antral follicle count (AFC), and tubal patency test (hysterosonosalpingography or hysterosalpingography) in case of AI. Further exploration may be required in specific cases.

Women presenting as single or as part of a heterosexual couple were excluded. In addition, couples who quit and did not undergo any kind of reproductive treatment were also excluded. Clinical data was retrieved from medical records in a pseudonymized manner. Variables retrieved included age, nationality, body mass index (BMI), previously known and newly diagnosed infertility factors, number of children, AMH levels, AFC, their intended treatment, the type of treatments finally performed, which patient was treated or which role each patient played, the outcome of the treatment, and the number of surplus frozen embryos.

The main outcome measure was cumulative live birth (LB) per treatment (which includes frozen embryo transfers in cases of IVF with own or donated oocytes and ROPA). The live birth rate (LBR) was defined as the number of deliveries resulting in at least one live born neonate. Live birth was defined as the delivery of ≥ 1 live newborn after 24 weeks of pregnancy.

The results are presented in absolute counts and proportions. For comparative analyses, the statistical tests used were the Student *t* -test (for normally distributed continuous variables), the Mann-Whitney U-test (for non-normally distributed continuous variables), and the chi-squared test (for categorical variables). Significance was defined with a 95% confidence interval (CI) ( *p* < 0.05). The main comparisons were made according to the first reproductive treatment performed (single versus double-parented method) and to the choices of the couples after medical counseling (patients who kept their intentions versus patients who changed their minds). All missing data were excluded from the analysis.

## Results

### Baseline Features

A total of 159 female couples had their first medical appointment at our clinic from January 2017 to December 2018, but 30 quit without beginning any ART. In the end, 129 couples were included ( Table 1 ). The mean age at the beginning of the first treatment was 36.2 years old, with a mean BMI of 23.7, a mean AMH value of 19.1 pmol/L, and a mean AFC of 13.4. Ninety-two percent of the patients had no previous children. Most patients were Spanish, but there were also patients from other European countries, Latin and North America, and Asia. Among the patients who chose a single-parent method (AI, IVF or donation of oocytes/embryos) as first treatment, the treated patients (versus their partners) were 1.4 years younger, with a lower proportion of women of advanced age (18.6% less) and 10.2 pmol/L more of AMH, but no significant differences were found regarding BMI, AFC or number of previous children. Regarding patients who began with ROPA, the receivers had a mean AFC 9.8 lower than the donors. No differences were found in the mean age, but there were no women with advanced age in the donor group. Only two patients submitted to ROPA, from different couples - one donor and one recipient – had previous children.

**Table 1:** Baseline features according to the type of first reproductive treatment performed

	Total	Single-parented methods ( *n* = 107 couples)			Double-parented method (ROPA) ( *n* = 22 couples)		
		Patient	Partner	* p*	Recipient	Donor	* p-value*
Mean age (years old)	36.6	35.1	36.4	0.03	34.6	32.2	0.15
Women with advanced age (percentage)	25%	18.5	37.1	0.003	19	0	0.04
Women without previous children (percentage)	91.9%	92.5	95.2	0.54	96	96	> 0.99
BMI	23.7	23.3	23.3	0.9	23.4	23.1	0.79
AFC	13.4	12.9	11.8	0.7	9.9	19.7	0.03
AMH (pmol/L)	19.1	23.4	13.4	0.02	17.7	23.2	0.59
Nationality	Spanish: 65% European (other): 31.8% Latin American: 2% Asian: 1.6% North American: 0.4%						

Abbreviations: AFC, antral follicle count; AMH, antimüllerian hormone; BMI, body mass index; ROPA, douple-parented method.

### Fertility

At the end of the medical workup, 46.1% of the couples had at least 1 member affected by ≥ 1 fertility-impairing condition. If advanced age (defined ≥ 40 years old) was added to the equation, this would make 68% of the patients. In 28.9% of these patients, at least 1 diagnosis was made during the medical workup by the fertility specialist. In the end, 32% of all the couples had no known condition potentially affecting fertility or advanced age.

Endometriosis was the leading condition among the previously known diagnoses (5% of the patients). On the other hand, low ovarian reserve (5.5% of the patients) and uterine disorders (4.7% of the patients) were the most frequent *de novo* diagnoses during the medical workup.

### Initial Intention and First Treatment

Before undergoing medical counseling, a large part of the couples had the intention to undergo AI (41%), with the others intending to undergo ROPA (26,4%) and IVF with own (17%) or donated oocytes/embryos (9.4%). Interestingly, 6.2% of the patients had no previous idea of the treatment they would choose.

After medical counseling, most of the couples willing to undergo AI or IVF did in fact undergo the desired treatment (only 11.3 and 9.1% ended up with another treatment as first line, respectively). On the other hand, a significantly higher percentage (38.2%; *p* = 0.005) of the couples willing to undergo ROPA as a first line treatment ended up undergoing a single-parented method – 20.6% opted for IVF and 17.6% for AI. The couples with no previous intention ended up undergoing AI or IVF with own oocytes ( Table 2 ).

**Table 2:** First reproductive treatments performed according to the previous intentions of the patients before the medical workup

		FIRST TREATMENT PERFORMED			
		AI (59) * n* (%)	IVF (36) * n* (%)	Donation (12) * n* (%)	ROPA (22) * n* (%)
Intended treatment before medical workup	Unknown (8)	4 (50)	4 (50)	-	-
	AI (47)	47 (88.7)	6 (11.3)	-	-
	IVF (22)	1 (4.5)	20 (91)	-	1 (4.5)
	Donation (12)	-	-	12 (100)	-
	ROPA (34)	7 (20.6)	6 (17.6)	-	21 (61.8)

Abbreviations: AI, artificial insemination. IVF, in vitro fertilization. ROPA, double-parented method.

*n* and percentage within the intended treatment group.

Interestingly, the percentage of couples who changed their mind was similar between the groups who had and had not a de novo diagnosis of a fertility issue during the medical workup (27 and 23%, respectively; *p* = 0.39).

In the end, 46% of the couples underwent AI as first treatment, 28% chose IVF with own oocytes, 16% chose ROPA, and 10% underwent IVF with donated oocytes/embryos.

### Treatments and Outcomes

**Fig. 1 (A and B):**
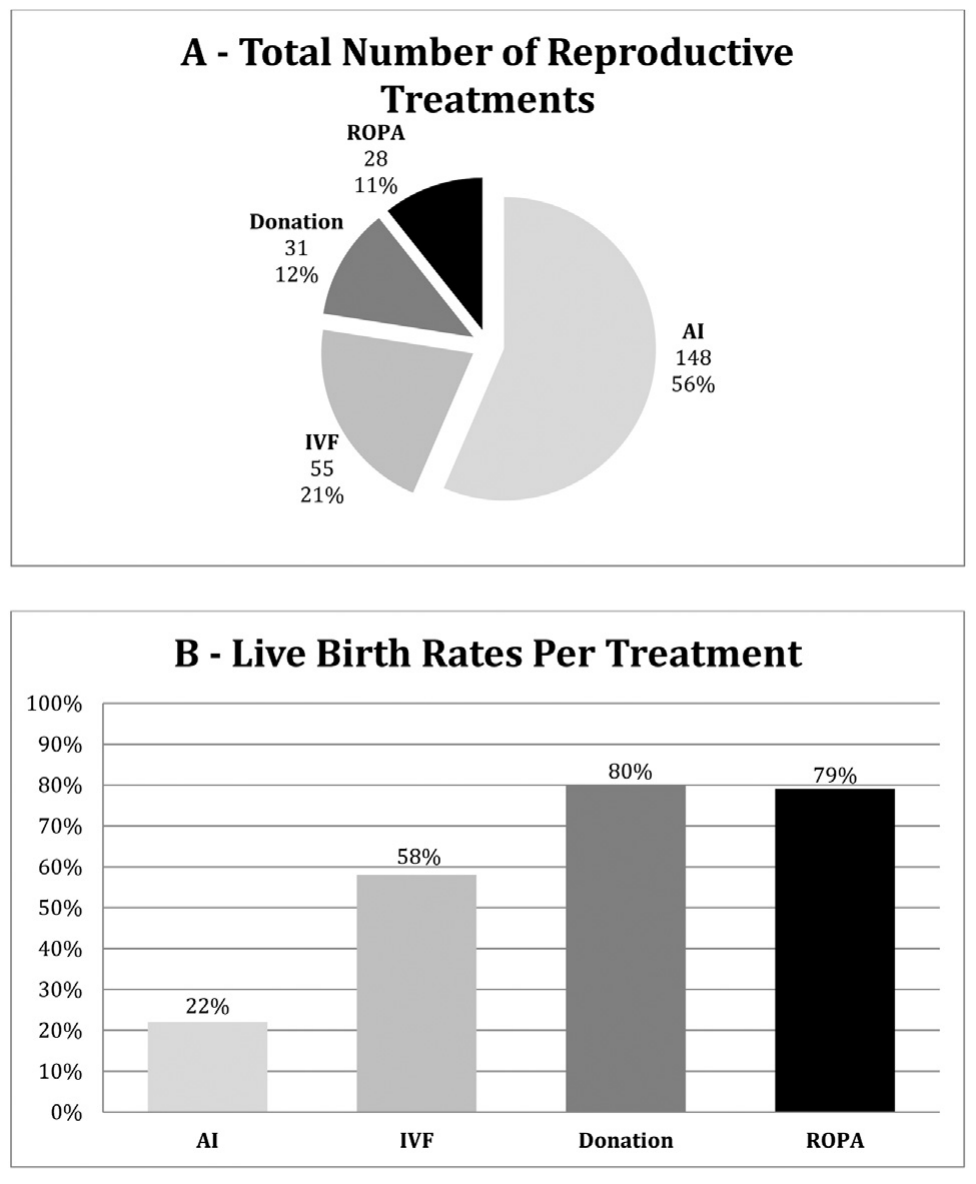
Reproductive treatments performed (1A: Total number of ART performed) (1B: Live birth rate of each type of treatment).

The total number of cycles performed was 148 AI, 55 IVF with own oocytes, 28 ROPA, and 31 IVF treatments with donated oocytes or embryos. The LBR per treatment (including frozen embryo transfers) was 80% for treatments with donated oocytes/embryos, with 79% for ROPA, 58% for IVF with own oocytes, and 22% for AI ( Figs. 1 A and B ).

### Live Births

Ninety-four out of the 129 couples (72.3%) achieved at least 1 LB – 55.8% of the couples had 1 LB, 16.3% had 2, and only 1 couple had 3 consecutive successful pregnancies. In 13% of the couples, both members of the couple gave birth. In the end, 120 children were born as a result of 115 LBs (5 twin pregnancies) – 37 (32.1%) by AI, 27 (23.5%) by IVF, 24 (20.8%) after ROPA, and 27 (23.5%) with donated oocytes or embryos.

### The Specific Case of ROPA

A total of 28 ROPA cycles were performed in 25 couples, which means that 3 couples underwent 2 consecutive ROPA cycles. All patients who underwent ROPA had already this intention since the beginning, and all of them had clearly the roles each one would play. Only 2 couples (8%) ended up inverting roles, under medical advice, due to newly diagnosed low ovarian reserve of one of the patients. The LBR per ROPA treatment (including frozen embryo transfers) was 79%. One of these successful pregnancies was a twin gestation. Most of these 22 couples who achieved a LB got pregnant after the first (45.5%) or the second (40.9%) embryo transfer; only a small minority needed 3 (4.5%) or 4 (9.1%) transfers to have a LB. In half of the cycles, the patients remained with surplus frozen embryos – a mean of 2.8 surplus embryos per cycle. None of the surplus embryos were used for a second pregnancy yet ( Table 3 ). Three couples underwent a second ROPA treatment. Two couples repeated the procedure with the same donor and receiver, one of them to have another child and the other following an unsuccessful first cycle. The latter only achieved pregnancy by transferring the last embryo to the donor mother due to repeated implantation failure, so in the end it was a single-parented IVF. The third couple opted for a reverse ROPA to change roles, despite having two surplus frozen embryos from the first treatment. We had no cases of reciprocal ROPA (two ROPAs at the same time, with both members playing both roles at the same time – a simultaneous “exchange” of embryos). All three second ROPA treatments were successful.

**Table 3:** Summary of ROPA treatments

ROPA	
Number of couples	25
Number of cycles	28 (22 as first reproductive treatment, 3 following another type of ART, 3 as second ROPA in the same couple)
Outcomes	
Number of live newborns	23
Live births (rate)	22 (79%)
Twin pregnancies	1 (4.5%)
Number of embryo transfers needed to achieve a live birth	
1	10 (45.5%)
2	9 (40.9%)
3	1 (4.5%)
4	2 (9.1)
Surplus embryos	
Number of couples with surplus embryos (percentage)	12 (48%)
Number of surplus embryos per couple (mean)	2.8
Couples who repeated ROPA	3
Case 1	Same-way ROPA for a second child (successful)
Case 2	Same-way ROPA after an unsuccessful first cycle, last embryo transferred to the donor mother (single-parented) due to recurrent implantation failure (successful)
Case 3	Reverse ROPA for a second child (successful)

Abbreviations: ROPA, double-parented method.

## Discussion

The mean age at the first reproductive treatment was 36 years old, which is consonant with that of the general population. In the most developed countries, during the last decades, women have progressively postponed maternity. As a result, the mean age of women resorting to reproductive treatments has gradually increased. 15 Nevertheless, unlike heterosexual couples, same-sex couples know *ad inicium* that they must resort to ART to reproduce, so they do not have the delay of unsuccessfully trying a spontaneous pregnancy. 16

As expected, as a general rule, priority was given to members of couples with the best prognosis to undergo the first treatment, which is reflected by a lower average age, with fewer cases of advanced age and higher levels of AMH, that is, a better ovarian reserve. Likewise, in cases of ROPA (double-parented method), oocyte donors tended to be younger and with greater ovarian reserve, although, in this case, data are not so reliable as a result of the smaller sample.

One of the main findings of the present study is that only nearly one third of the couples had absolutely no condition impairing fertility nor advanced age. Almost half (47%) of the couples had at least 1 member affected by an organic fertility disorder. This finding is quite intriguing since lesbian couples are usually regarded as fertile patients. Nevertheless, one must keep in mind that this is not a synonym of infertility. Again, unlike heterosexual couples, most of these patients had never tried natural conception. 14 17 These disorders were discovered during an exhaustive medical workup, so they could eventually be subclinical and never have a real impact on fertility.

Most couples were already decided about their treatment of choice. Only a small part of the couples who wanted AI or IVF ended up undergoing a different treatment. On the other hand, a significantly higher percentage of couples looking for ROPA ended up undergoing a single-parented method. Our impression is that this was mainly due to economic reasons, even though we were not able to validate this fact. The discovery of a new diagnosis could also be the reason for changing treatment. In fact, a slightly higher but nonsignificant percentage of new diagnoses of fertility disorders was found in the group of patients who changed the first line treatment after the medical workup and counseling.

Regarding the outcomes, we opted for using the LBR per cycle as our main outcome. It must be considered that, using a rate per cycle, a successful IVF cycle may include several embryo transfers. In addition, unlike many studies in the field of reproductive medicine that report gestational rates, we chose LBR to consider pregnancy losses. 18

The LBRs for all treatments were considerably high, which is probably a consequence of ours being a population with no infertility background. Almost three in four couples had at least one LB. Most of them did not undergo further treatments after having their first child. Nevertheless, one must keep in mind that a maximum of 3 years have passed since the first LB, which means that some couples may still return for another child. These results are in line with previous studies that report better outcomes after reproductive treatments in female couples compared with heterosexual couples. 5 16 17

Concerning the ROPA method, 21% of the LBs resulted from this double-parented method. This means that four in five children were biologically related only to one of their mothers (as a result of single-parented methods). We found a higher LBR in ROPA compared with that of single-sided IVF (79 versus 58%). This could be explained by the fact that ROPA offers the possibility of choosing the best of each side; in other words, if one of the patients has an ovarian condition and her partner has an uterine disorder, the former may play as receiver and the other as donor. 3 4 5 In fact, two couples ended up inverting roles under medical advice due to low ovarian reserve. In addition, we had a case of several unsuccessful embryo transfers, probably due to impaired endometrial receptiveness, which was solved simply by changing the receiving mother. Although it was no longer a double-parented method, this case reflects the flexibility of this method and the range of options it offers, which culminate in good success rates. Interestingly, in the end, the LBR of ROPA was almost as high as that of IVF with donated oocytes or embryos, in which embryos are of good quality because they come from young, selected donors.

Regarding conventional IVF, most couples had surplus embryos after achieving pregnancy. On the one hand, this facilitates the process of having a second child. However, some couples may want to switch roles when having a second child. This may pose an important ethical dilemma when it comes to create new embryos in the presence of frozen surplus ones. 19 This was the case of one of ours couple who underwent a reverse ROPA to have a second child, despite having two good quality frozen embryos.

The present study has its limitations, such as the reduced sample size, the short time spent since the treatments were performed, its retrospective and merely descriptive nature, and the fact that it was based on a single private clinic, in which the economic factor may eventually condition the choices of the couples.

## Conclusion

The present study highlights the importance of a thorough medical workup in female couples resorting to ART, since a large proportion of these patients may present a fertility disorder and may want or need to change their plans after adequate medical counseling. Despite these facts, overall, female couples may be considered of good reproductive prognosis and expect a high probability of having a child. Many couples end up opting for a single-parented method, despite the growing availability of the ROPA method. The ROPA method offers not only the possibility for women to share biological motherhood with good success rates, but it can also be a good alternative when there is some factor limiting fertility, due to its flexibility and range of options within the couple.
